# Measurement Invariance of a Latent Dementia Index by Gender in the Aging, Demographics, and Memory Study

**DOI:** 10.1093/geroni/igab046.3624

**Published:** 2021-12-17

**Authors:** Joseph Saenz, Alice Kim, Christopher Beam

**Affiliations:** 1 UNIVERSITY OF SOUTHERN CALIFORNIA, Los Angeles, California, United States; 2 University of Southern California, University of Southern California, California, United States

## Abstract

Population-based aging studies allow researchers to study dementia and its correlates. Few include dementia diagnoses. Latent variable models have been used to create latent dementia indexes (LDI) using cognitive and functional ability to approximate dementia. The LDI is applied across diverse populations, but it is unclear whether gender affects its measurement properties. We assess whether the LDI can be used to measure dementia equivalently for men and women. We use the 2001-2003 Aging, Demographics, and Memory Study (n=856, 355 men, 501 women). Cognitive ability was assessed using memory, executive function, attention, spatial ability, orientation, and language tasks. Functional ability was informant-reported. We used confirmatory factor analysis to test factorial invariance across gender and compare latent means to determine which group had lower means, consistent with greater dementia likelihood. Model fitting results suggest metric invariance of the LDI but only partial scalar invariance across gender. Latent mean differences in the LDI were observed (Mdiff = .39, SE = 0.19, p = .042), with women lower, on average, than men. Correlations between LDI and dementia diagnosis were stronger for both men (r=-.82) and women (r=-.85) than correlations between dementia and Mini-Mental Status Exam scores (-.69 and -.73, respectively). The LDI may be reliably and validly used to measure and compare dementia likelihood in men and women. Results suggest lower LDI scores in women, indicating greater dementia likelihood. Gender differences may be partially attributed to differences in measurement properties of items, possibly due to gender differences in educational returns and employment factors.

